# Supplementary material for: The nucleocytosolic O-fucosyltransferase SPINDLY affects protein expression and virulence in Toxoplasma gondii
Source: J Biol Chem. 2020 Nov 23;296:100039. doi: 10.1074/jbc.RA120.015883 (PMC7949088; doi:10.1074/jbc.RA120.015883)
Supplement: Tables and Figures [file mmc1.docx]

Supporting information

**The nucleocytosolic *O*-fucosyltransferase *Spindly* affects protein expression and virulence in *Toxoplasma gondii***

Giulia Bandini^1,*,∮^, Carolina Agop-Nersesian^1^ Hanke Van der Wel^2^, Msano Mandalasi^2,3^, Hyun W. Kim^2^, Christopher M. West^2,3,4^, John Samuelson^1^

^1^Department of Molecular and Cell Biology, Boston University Henry Goldman School of Dental Medicine, Boston, MA 02118

^2^Department of Biochemistry and Molecular Biology, ^3^Center for Tropical and Emerging Global Diseases, ^4^Complex Carbohydrate Research Center, University of Georgia, Athens GA 30602

^∮^Current address: Department of Biology, University of York, York, UK

^*^ corresponding author

This Supporting Information file includes:

**Table S1**

**Figures S1**

**Table S1**. Primers used in this study

| **Primer** | **Number** | **Sequence** | **Restriction sites** | **Ref.** |
| --- | --- | --- | --- | --- |
| gmdF | P101 | CGGATCCAAAATGGAAGGCGAAAACG |  |  |
| gmdR | P102 | CCTAGGCGCCTTTTGGACTCTCC |  |  |
| SPYt2_F | P127 | AAGTTGAGCTGCTAGCTCGCAAACACG |  |  |
| SPYt2_R | P128 | AAAACGTGTTTGCGAGCTAGCAGCTCA |  |  |
| SPYKO_RO_F | P129 | AGGCCTCACGCCGGAGTCTCCTCCTCACAGTCGCGGCCGTTCtcgaaggctgctagtactg | StuI |  |
| SPYKO_RO_R | P130 | GGCGCCAGACTCTTGATCGCCTCTGCAGCGCTCTGGCTgcttaacaccattgcattcc | SfoI |  |
| TgSPY_F | P161 | GAATGGACCTGATCCTTCC |  |  |
| TgSPYe3_R | P162 | TGGTTGGTGAGGATTCG |  |  |
| K793A_F | P173 | CAACCTGGCGgcaCTGGGTAACC |  |  |
| K793A_R | P174 | TTGAAGCTACCGAAGGTAAC |  |  |
| H623A_F | P175 | CTTCTTCACCgcaAGCGTCAGCTACTTCATCC |  |  |
| H623A_R | P176 | TCCGGACCAATGTAGCCA |  |  |
| D619A_F | P177 | CATTGGTCCGgcaTTCTTCACCCACAG |  |  |
| D619A_R | P178 | TAGCCAACGCGGATCACA |  |  |
| 3T_F | P179 | GCGGAAGCGTACAACAAC |  |  |
| 3T_R | P180 | CATAGATCTAAAAGGGAATTCAAGAAAAAATG |  |  |
| gt41_F | P181 | AACGCGTTCCACAACAAAC |  |  |
| gt41_R | P182 | CATAGATCTAAAAGGGAATTCAAG |  |  |
| gt41_dia_R | P197 | GCAGGGTCTTGAACATCTG |  |  |
| tgmut_F | P198 | CCAGGGTGACATCGAGGACA |  |  |
| 273500_F2 | P206 | ATGAATGGACCTGATCCTTCCC |  | 31 |
| 273500_R | P207 | CTAGCAGCTGGCGATACAGC |  | 31 |
| DHFR_seqR | P208 | CGAATGACACACAGGAACTACGC |  | 31 |
| E692K_F | P226 | CATTCTGGTCAAGCTGGCAGGTC |  |  |
| E692K_R | P227 | TCGATACGGTCTTCCTCG |  |  |
| G695D_F | P228 | GAACTGGCAGaTCACACCGCA |  |  |
| G695D_R | P229 | GACCAGAATGTCGATACGGTC |  |  |
| 720F | P233 | GAGCAACATGCTGTACGCAT |  |  |
| Hpt_gRNAF | P300 | AAGTTGGCACGATAGCCCCTTCATTG |  |  |
| Hpt_gRNAR | P301 | AAAACAATGAAGGGGCTATCGTGCCA |  |  |
| GFP_f | P35 | AGCTCGAGATGAGTAAAGGAGAAGAAC | XhoI |  |
| GFP_r | P36 | GCTTAATTAACTATTTGTATAGTTCATCCATG | PacI | 21 |
| SRD_F | P7 | CTAAAATGGAGACCTCTTCTC |  | 22 |
| cMycR | P8 | CACCGTTCAAGTCTTCCTCGG |  | 22 |

| *E. coli* |  |  |  |  |
| --- | --- | --- | --- | --- |

| Tg-D619A-Q5S |  | GCTACATTGGgcccgcCTTCTTCACCCAC | ApaI |  |
| --- | --- | --- | --- | --- |
| Tg-D619A-Q5AS |  | CAACGCGGATCACACGGC |  |  |
| Tg-H623A-Q5S1 |  | CTTCTTCACCgctAGCGTCAGCTACTTCATC | NheI |  |
| Tg-H623A-Q5AS |  | TCCGGACCAATGTAGCCA |  |  |
| Tg-E692K-Q5S |  | CATTCTGGTCaagcttGCAGGTCACAC | HindIII |  |
| Tg-E692K-Q5AS |  | TCGATACGGTCTTCCTCG |  |  |
| Tg-G695D-Q5S |  | cagaTCACACCGCACACAACCG | NheI(split) |  |
| Tg-G695D-Q5AS |  | ctagcTCGACCAGAATGTCGATACG | NheI(split) |  |
| Tg-K793A-Q5S |  | CAACCTGGCGgccctaGGTAACCAGG | AvrII |  |
| Tg-K793A-Q5AS |  | TTGAAGCTACCGAAGGTAAC |  |  |

**FIGURES**

**Figure S1. *Tg*SPY cDNA sequence.** Predicted native coding sequence, based on TGME49_273500, is shown in black; *E. coli* codon optimized (synthetic) sequence, which includes cDNA sequence and 3’ UTR, after cloning in pET15-TEV is shown in blue; calculated amino acid sequence of the synthetic cDNA is in bold black. The native start and stop codons are highlighted in teal green. Restriction sites are marked in green (Bgl*II*), blue (Nco*I*), grey (Nde*I*), yellow (BamH*I*), pink (Spe*I*), and purple (Hind*III*).

**M G S S H**

gatatacc**atg**ggcagcagccat

**H H H H H S S G R E N L Y F Q G H M R S**

catcatcatcatcacagcagcggcagagaaaacttgtatttccagggccaTATGAGATCT

**M N G P D P S R P C L T P E S P P H S R 20**

ATGAATGGACCTGATCCTTCCCGGCCGTGCCTCACGCCGGAGTCTCCTCCTCACAGTCGC 60 *Tg*spy

ATGAACGGTCCGGACCCGTCTCGTCCGTGCCTGACCCCGGAGAGCCCGCCGCACAGCCGT synthetic

**G R S C P P S D Q L G N S S S S S A T T 40**

GGCCGTTCCTGCCCTCCCTCGGATCAACTTGGCAATTCCTCTTCGTCTTCTGCTACCACA 120

GGCCGCAGCTGCCCGCCGAGCGACCAGCTGGGTAACAGCAGCAGCAGCAGCGCAACCACC

**P A T D L D S A V Q D N I F A Q A P S E 60**

CCGGCAACCGACTTGGACTCTGCAGTGCAGGACAACATCTTTGCGCAGGCGCCCTCGGAA 180

CCGGCAACCGACCTGGACAGCGCGGTGCAGGACAACATCTTCGCACAGGCACCGAGCGAA

**T S S R P L P I P A A N A L R H L P S P 80**

ACTTCTTCTCGTCCTCTTCCGATCCCTGCAGCCAACGCCCTGCGACACCTGCCTTCGCCG 240

ACCAGCAGCCGTCCGCTGCCGATTCCGGCAGCGAACGCGCTGCGTCACCTGCCGAGCCCG

**P L A R E A C D G G A R P S P Q R N C C 100**

CCGCTCGCCAGGGAAGCCTGTGACGGAGGCGCGAGGCCCTCGCCTCAGCGGAACTGCTGC 300

CCGCTGGCACGTGAGGCGTGCGACGGCGGTGCGCGTCCGAGCCCGCAGCGCAACTGCTGC

**D G R P A A C N H R A V S D V C A S L P 120**

GACGGCAGGCCGGCTGCATGCAACCACCGTGCAGTCAGCGACGTGTGTGCGTCGCTCCCA 360

GACGGCCGTCCGGCGGCGTGCAACCACCGTGCAGTTAGCGACGTCTGCGCAAGCCTGCCG

**E K S F A R H F L T S S G T F P S A A E 140**

GAAAAGTCCTTTGCTCGTCATTTCCTCACGTCTTCTGGGACCTTTCCGTCTGCTGCGGAG 420

GAAAAGAGCTTCGCGCGTCACTTCCTGACCAGCAGCGGTACCTTCCCGAGCGCAGCAGAG

**I L K K A A F F N S G N R P H D A L L L 160**

ATCCTAAAGAAAGCGGCGTTCTTCAACTCGGGCAACCGCCCTCATGATGCCCTTCTCCTC 480

ATCCTGAAGAAAGCGGCGTTCTTCAACAGCGGTAACCGTCCGCACGACGCACTGCTGCTG

**C N A G L E V Y A E D A D L W N C K G V 180**

TGCAACGCTGGCCTGGAAGTTTACGCCGAAGATGCCGACTTGTGGAACTGCAAAGGCGTC 540

TGCAACGCGGGTCTGGAAGTGTACGCGGAAGACGCGGACCTGTGGAACTGCAAAGGCGTG

**T L R A L G R L Q E A L D C C R E A L R 200**

ACTCTTCGAGCTCTTGGAAGACTCCAGGAGGCACTTGATTGCTGCCGAGAGGCGCTTCGT 600

ACCCTGCGTGCGCTGGGTCGCCTGCAGGAAGCGCTGGACTGCTGCCGTGAGGCACTGCGT

**L D P G N T N A L N N I G V A L K E R G 220**

CTTGATCCAGGGAATACAAATGCTCTAAACAACATTGGAGTTGCATTGAAGGAGAGAGGC 660

CTGGACCCGGGCAACACCAACGCGCTGAACAACATCGGCGTTGCGCTGAAGGAGCGTGGT

**E L L Q A V E H Y R A S L V A N P H Q P 240**

GAACTTCTACAAGCTGTCGAGCACTACCGGGCCTCTCTGGTGGCGAATCCTCACCAACCA 720

GAACTGCTGCAGGCGGTCGAACACTACCGTGCAAGCCTGGTGGCGAACCCGCACCAGCCG

**T C R T N L A V A L T D L G T K L K Q E 260**

ACATGCCGCACAAACTTGGCAGTTGCCCTCACCGACTTGGGCACGAAGCTGAAGCAGGAG 780

ACCTGCCGCACCAACCTGGCAGTTGCACTGACCGACCTGGGCACCAAGCTGAAACAGGAA

**K K L Q A A L V C Y T E A L T A D P T Y 280**

AAGAAGCTGCAGGCAGCGCTCGTTTGCTACACAGAGGCACTGACCGCAGACCCGACCTAC 840

AAGAAACTGCAGGCAGCACTGGTGTGCTACACCGAAGCACTGACCGCGGACCCGACCTAC

**A P C Y Y N L G V I H A E T D D P H T A 300**

GCGCCTTGCTACTACAACCTTGGCGTTATCCACGCAGAAACAGATGACCCCCACACCGCT 900

GCGCCGTGCTACTACAACCTGGGTGTGATTCACGCAGAGACCGACGACCCGCACACCGCG

**L Q M Y R E A T R L N P S Y V E A Y N N 320**

CTCCAGATGTACAGAGAAGCCACGCGCCTCAATCCCAGCTACGTCGAGGCTTACAACAAC 960

CTGCAGATGTACCGTGAAGCGACCCGCCTGAACCCGAGCTACGTGGAGGCGTACAACAAC

**M G A V C K N L G K L E D A I S F Y E K 340**

ATGGGCGCTGTGTGTAAGAACCTAGGCAAGCTGGAAGACGCTATTTCGTTCTATGAGAAG 1020

ATGGGCGCGGTTTGCAAAAACCTGGGTAAACTGGAAGACGCGATTAGCTTCTACGAGAAA

**A L A C N A N Y Q M S L S N M A V A L T 360**

GCCCTTGCATGCAATGCAAACTACCAGATGAGTCTGAGCAACATGGCTGTTGCTCTCACC 1080

GCGCTGGCGTGCAACGCGAACTACCAGATGAGCCTGAGCAACATGGCAGTGGCGCTGACC

**D L G T Q Q K A S E G A K K A I S L Y K 380**

GACCTTGGAACTCAGCAGAAAGCTTCTGAAGGCGCGAAGAAGGCAATTTCGCTCTACAAA 1140

GACCTGGGCACGCAGCAGAAGGCAAGCGAGGGTGCGAAGAAAGCGATCAGCCTGTACAAG

**K A L I Y N P Y Y S D A Y Y N L G V A Y 400**

AAGGCCTTAATTTACAATCCGTATTATTCGGATGCGTACTACAATCTGGGCGTCGCGTAC 1200

AAAGCGCTGATTTACAACCCGTACTACAGCGACGCGTACTACAACCTGGGCGTTGCGTAC

**A D L H K F D K A L V N Y Q L A V A F N 420**

GCCGACTTGCACAAATTCGACAAGGCACTGGTGAACTATCAGCTGGCAGTGGCCTTCAAT 1260

GCGGACCTGCACAAATTCGACAAGGCGCTGGTTAACTACCAGCTGGCAGTTGCATTCAAC

**P R C A E A Y N N M G V I H K D R E N T 440**

CCTCGATGTGCTGAGGCGTACAACAACATGGGGGTCATCCATAAGGATAGAGAAAACACG 1320

CCGCGTTGCGCGGAAGCGTACAACAACATGGGTGTTATCCACAAAGACCGCGAGAACACC

**D Q A T V Y Y N K A L E I N P D F S Q T 460**

GACCAGGCCACTGTTTACTACAACAAAGCTCTGGAAATAAATCCGGACTTCTCTCAAACG 1380

GACCAGGCGACCGTCTACTACAACAAGGCGCTGGAAATTAACCCGGACTTCAGCCAGACC

**L N N L G V L Y T C T G K I G E A L H F 480**

CTCAATAACCTTGGCGTGCTCTACACGTGCACCGGGAAGATTGGCGAGGCGCTGCACTTT 1440

CTGAACAACCTGGGCGTTCTGTACACCTGCACCGGCAAAATCGGCGAGGCGCTGCACTTC

**A K R A I E V N P N Y A E A Y N N L G V 500**

GCAAAGCGTGCTATTGAAGTCAATCCGAACTACGCCGAGGCATACAACAACTTGGGAGTT 1500

GCGAAGCGTGCGATTGAAGTCAACCCGAACTACGCGGAGGCGTACAACAACCTGGGCGTG

**L Y R D Q G D I E D S V K A Y D K C L L 520**

CTGTACCGGGACCAGGGCGATATCGAGGACTCTGTGAAAGCGTATGACAAATGCTTGCTT 1560

CTGTACCGCGACCAGGGTGACATCGAGGACAGCGTTAAAGCGTACGACAAGTGCCTGCTG

**L D P N S P N A F H N K L L A L N Y L E 540**

CTGGATCCCAACTCACCAAACGCCTTCCACAACAAGTTACTGGCGTTGAACTATTTGGAG 1620

CTGGACCCGAACAGCCCGAACGCGTTCCACAACAAACTGCTGGCGCTGAACTACCTGGAA

**N L P E N E I C R V S E K W G L H F L S 560**

AATTTGCCTGAAAACGAGATATGTCGCGTTTCTGAGAAGTGGGGACTGCATTTCCTTTCT 1680

AACCTGCCGGAGAACGAAATCTGCCGTGTTAGCGAGAAGTGGGGTCTGCACTTCCTGAGC

**S R S P Y T S W L C P P V T I S P A L P 580**

TCTCGGTCTCCGTACACTTCCTGGCTGTGTCCGCCGGTCACCATCTCACCTGCCCTGCCT 1740

AGCCGCAGCCCGTACACCAGCTGGCTGTGCCCGCCGGTTACCATTAGCCCGGCACTGCCG

**S S A V R S P A R P S S S S A S S S P A 600**

TCGTCTGCCGTCCGCTCACCTGCCCGTCCATCTTCATCGTCAGCTTCTTCGTCTCCTGCC 1800

AGCAGCGCGGTCCGTAGCCCGGCGCGCCCGAGCAGCAGCAGCGCAAGCAGCAGCCCGGCA

**S P G D S S A S R V I R V G Y I G P D F 620 D619A** TCTCCTGGCGATTCTTCAGCTTCAAGAGTCATACGCGTGGGGTACATTGGACCAGATTTC 1860

AGCCCGGGTGACAGCAGCGCGAGCCGTGTGATCCGCGTTGGCTACATTGGTCCGGACTTC

**F T H S V S Y F I H A P L V Y H D K A K 640 H623A** TTTACTCATTCTGTCTCTTACTTCATCCATGCTCCCTTGGTCTACCACGACAAAGCGAAG 1920

TTCACCCACAGCGTCAGCTACTTCATCCACGCGCCGCTGGTGTACCACGACAAGGCGAAA

**F H I T V Y A N V I R E D E K T Q M F K 660**

TTCCACATCACTGTCTACGCGAACGTCATCCGAGAAGACGAAAAGACTCAAATGTTCAAG 1980

TTCCACATCACCGTTTACGCGAACGTCATTCGTGAGGACGAAAAAACCCAGATGTTCAAG

**T L P H R W R S I V G L N E Q E V A R I 680**

ACGCTCCCGCACCGCTGGCGGTCTATCGTGGGGTTGAACGAGCAGGAGGTTGCTCGGATC 2040

ACCCTGCCGCACCGTTGGCGCAGCATTGTCGGCCTGAACGAGCAGGAAGTGGCGCGTATC

**I R E E D R I D I L V E L A G H T A H N 700 E692K,G695D** ATCCGAGAAGAAGACCGAATCGACATTTTGGTGGAACTCGCAGGGCACACAGCGCACAAC 2100

ATTCGCGAGGAAGACCGTATCGACATTCTGGTCGAACTGGCAGGTCACACCGCACACAAC

**R L D V M A C K P A P V Q I S W I G Y P 720**

CGCCTCGACGTGATGGCGTGCAAACCTGCGCCGGTTCAGATCAGCTGGATTGGCTATCCG 2160

CGTCTGGACGTTATGGCGTGCAAACCGGCGCCGGTCCAGATCAGCTGGATTGGCTACCCG

**N T T G L K T I D F R I T D A V A D P L 740**

AACACAACCGGCTTGAAGACCATCGACTTCCGCATCACTGACGCCGTCGCGGACCCGCTG 2220

AACACCACCGGTCTGAAGACCATCGACTTCCGTATTACCGACGCAGTGGCGGACCCGCTG

**T T T E R Y V E E L V R M P N C F L C Y 760**

ACCACAACGGAGAGGTATGTGGAAGAGCTGGTCCGCATGCCCAACTGCTTCCTCTGCTAC 2280

ACCACCACCGAGCGTTACGTGGAGGAACTGGTTCGCATGCCGAACTGCTTCCTGTGCTAC

**Q P P P D F P K H V P A K P P P V L D H 780**

CAGCCGCCTCCCGACTTCCCGAAGCATGTGCCAGCGAAGCCGCCCCCCGTTCTCGATCAC 2340

CAGCCGCCGCCGGACTTCCCGAAACACGTTCCGGCAAAGCCGCCGCCGGTCCTGGACCAC

**G V V T F G S F N N L A K L G N Q V I E 800 K793A** GGCGTCGTCACCTTCGGCTCGTTCAACAATCTCGCAAAACTCGGCAATCAGGTGATTGAG 2400

GGCGTGGTTACCTTCGGTAGCTTCAACAACCTGGCGAAACTGGGTAACCAGGTGATCGAA

**I W S R I L N A V P N S R L L V K A R P 820**

ATCTGGAGTCGGATTTTGAATGCCGTCCCGAATAGCCGGTTGCTGGTCAAAGCCCGGCCG 2460

ATTTGGAGCCGCATCCTGAACGCGGTGCCGAACAGCCGTCTGCTGGTTAAAGCGCGCCCG

**F A N K E M Q R K F K A K F E A H G I S 840**

TTTGCAAACAAGGAAATGCAGCGGAAATTCAAGGCGAAATTTGAAGCGCATGGAATCTCG 2520

TTCGCGAACAAGGAAATGCAGCGTAAGTTCAAAGCGAAGTTCGAGGCGCACGGCATCAGC

**G D R I D A M A L I P A C M D H L M V Y 860**

GGCGATCGTATCGACGCCATGGCTCTCATTCCTGCATGCATGGATCACTTAATGGTCTAT 2580

GGTGACCGCATTGACGCGATGGCGCTGATCCCGGCGTGCATGGACCACCTGATGGTCTAC

**S L V D I A L D S F P Y A G T T T T C E 880**

TCGCTAGTTGACATCGCCCTCGATTCCTTCCCTTACGCCGGAACAACAACGACCTGTGAG 2640

AGCCTGGTGGACATTGCACTGGACAGCTTCCCGTACGCGGGTACCACCACCACCTGCGAG

**A L V M G V P V V S L R R P N I H A H N 900**

GCACTTGTCATGGGCGTCCCTGTCGTCTCTCTTCGTCGCCCAAACATCCACGCACACAAT 2700

GCACTGGTCATGGGTGTGCCGGTTGTGAGCCTGCGTCGCCCGAACATTCACGCACACAAC

**V G A T L L V N Y G L P E L I A D D P E 920**

GTAGGAGCAACTCTGCTGGTTAACTACGGACTGCCCGAACTTATCGCAGACGATCCCGAA 2760

GTGGGTGCAACCCTGCTGGTTAACTACGGTCTGCCGGAACTGATTGCGGACGACCCGGAG

**Q Y V R V A V E L A G D V E R L K R Y R 940**

CAATATGTTCGTGTAGCTGTCGAGCTCGCAGGGGATGTCGAGCGCCTAAAACGCTATCGG 2820

CAGTACGTTCGTGTTGCAGTGGAACTGGCAGGTGACGTGGAGCGTCTGAAGCGTTACCGC

**Q S I R E S V L E K A S E P H A K Q F T 960**

CAGAGCATCCGCGAATCAGTTCTCGAGAAGGCGTCTGAGCCGCATGCGAAGCAGTTCACT 2880

CAGAGCATCCGCGAGAGCGTTCTGGAAAAAGCGAGCGAGCCGCACGCGAAGCAGTTCACC

**R D L E E L Y R Q L L A R K H R Q K P R 980**

CGCGACTTAGAGGAGCTGTATCGCCAGCTGCTAGCTCGCAAACACCGGCAAAAGTAG 2937

CGTGACCTGGAGGAACTGTACCGCCAGCTGCTGGCGCGTAAACACCGCCAGAAGCCTAGG

*****

TAAggatcctaataactaagtaaactagtgctgagcaataactagcataaccccttgggg

cctctaaaggcccggcagtaccggcataaccaagcctatgccTacagcatccagggtgac

ggtgccgaggatgacgatgagcgcattgttagatttcatacacggtgcctgactgcgtta

gcaatttaactgtgataaactaccgcattaaagcttatcgatgataagctgtcaaacatg

agaa
